# Comparative Efficacy and Safety of Extended Versus Standard Interval Dosing of Natalizumab in Relapsing–Remitting Multiple Sclerosis Patients: A Multicenter Analysis

**DOI:** 10.1111/cns.70445

**Published:** 2025-05-19

**Authors:** Meral Seferoğlu, Abdulkadir Tunç, Ali Özhan Sıvacı, Bilge Piri Çınar, Sena Destan Bünül, Özlem Ethemoğlu, Ülgen Yalaz Tekan, Mehmet Fatih Yetkin

**Affiliations:** ^1^ Department of Neurology University of Health Sciences Bursa Yuksek Ihtisas Training and Research Hospital Bursa Turkey; ^2^ Department of Neurology, Faculty of Medicine Sakarya University Sakarya Turkey; ^3^ Department of Neurology, Faculty of Medicine Samsun University Samsun Turkey; ^4^ Department of Neurology, Faculty of Medicine Kocaeli University Kocaeli Turkey; ^5^ Department of Neurology, Faculty of Medicine Harran University Urfa Turkey; ^6^ Clinic of Neurology Şişli Hamidiye Etfal Training and Research Hospital Istanbul Turkey; ^7^ Department of Neurology, Faculty of Medicine Erciyes University Kayseri Turkey

**Keywords:** drug administration schedule, magnetic resonance imaging, natalizumab, relapsing–remitting multiple sclerosis, treatment adherence and compliance

## Abstract

**Background:**

Extended interval dosing (EID) of natalizumab (NTZ) every 6 weeks may reduce adverse events while maintaining efficacy. This study compared the effectiveness and safety of EID versus standard interval dosing (SID) in relapsing–remitting multiple sclerosis (RRMS) patients, focusing on treatment adherence and its impact on clinical and radiological outcomes.

**Methods:**

This retrospective study involved 80 patients with RRMS from seven clinics: 52 received SID (300 mg every 4 weeks), and 28 received EID (300 mg every 6 weeks). Clinical and radiological disease activity, treatment adherence, and adverse events were assessed.

**Results:**

The SID and EID groups differed significantly in sex distribution (78.8% female in SID vs. 46.4% in EID, *p* = 0.007), but median age was similar (32 vs. 36 years, *p* = 0.209). Clinical and radiological worsening rates were similar between the groups, with no significant differences (combined worsening: 9.6% in the SID group vs. 17.9% in the EID group, *p* = 0.308; radiological worsening: 5.8% in the SID group vs. 7.1% in the EID group, *p* = 1.00; clinical worsening: 9.6% in the SID group vs. 10.7% in the EID group, *p* = 1.00). Adherence rates were comparable across both dosing regimens, and no significant differences were observed in terms of treatment discontinuation. No progressive multifocal leukoencephalopathy cases were reported.

**Conclusion:**

Both SID and EID provide comparable efficacy and safety profiles, with similar adherence rates. Despite the observed sex distribution imbalance, additional analyses confirmed no significant sex‐ or group‐related differences in baseline disability or clinical worsening, strengthening the interpretation that EID preserves efficacy. Findings should still be interpreted with caution due to the study's retrospective nature and limited sample size.

## Graphical abstract

This study compares Extended Interval Dosing (EID) and Standard Interval Dosing (SID) of Natalizumab in RRMS patients. The findings demonstrate comparable clinical and radiological efficacy, similar adherence rates, and no reported PML cases in both groups, supporting EID as a flexible and safe alternative for long‐term treatment.

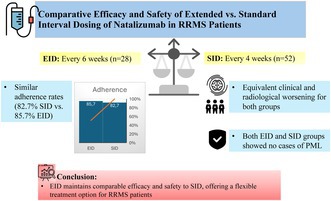

## 1 Introduction

Natalizumab (NTZ), a humanized monoclonal antibody, has emerged as a pivotal therapy in the management of relapsing–remitting multiple sclerosis (RRMS). This medication targets the α4‐subunit of α4β1‐integrin on leukocytes, thereby inhibiting the migration of these cells into the central nervous system, which is a key factor in the pathogenesis of RRMS [1, 2]. Natalizumab's standard interval dosing (SID) regimen, involving 300 mg every 4 weeks, has been demonstrated to effectively reduce both the clinical and radiological activity of MS, significantly impacting the progression of the disease [3, 4].

Nevertheless, the utilization of NTZ carries inherent hazards. A major concern associated with its use is the increased risk of progressive multifocal leukoencephalopathy (PML), an uncommon but potentially lethal cerebral infection caused by John Cunningham virus (JCV) [5, 6]. Patients who have received NTZ treatment for more than 2 years, who have tested positive for JCV antibodies, or who have a history of immunosuppressive therapy are particularly at heightened risk of developing PML [7, 8]. To address these hazards, a method called extended interval dosing (EID) of NTZ has been suggested and is becoming more widely accepted in clinical practice. This involves administering the medication every 6 weeks. The objective of EID is to mitigate the potential for PML while preserving the effectiveness of the drug [9, 10]. This approach is based on the hypothesis that partial desaturation of drug receptors could allow a degree of antiviral immune response restoration, potentially lowering the risk of PML [11]. While retrospective studies have indicated that EID may not compromise the efficacy of NTZ, prospective data to support this are still limited [12]. Another proposed modification to conventional dosing is interval therapy, which involves planned annual treatment interruptions. In a prospective study by Berkovich et al., an annual 12‐week interruption of NTZ was shown to be well tolerated and was not associated with increased relapse rates, disability progression, or MRI activity. The observed reversal of CD4+ T‐cell redistribution during the interruption highlights the immunological responsiveness to NTZ pharmacodynamics and provides further support for flexible treatment strategies in selected patients [13]. This growing body of evidence reflects a need to better understand the implications of different NTZ dosing approaches, particularly with regard to safety, immune surveillance, and patient adherence.

In this study, we sought to explore the effectiveness and safety of EID in comparison with those of SID, with a particular focus on treatment adherence and the factors influencing disease activity in patients with RRMS. We aim to contribute valuable data to the ongoing discourse on the optimal dosing regimen of NTZ in the management of RRMS, a topic of critical importance given the balance that must be struck between efficacy and safety in long‐term treatment strategies.

## 2 Materials and Methods

### 2.1 Study Design and Patient Selection

This retrospective study was conducted in seven MS clinics. Institutional review board approval was secured, ensuring adherence to ethical guidelines for human research (Application no: 2024‐TBEK 2024/02‐1). The study population consisted of patients aged 18–55 years with a confirmed diagnosis of RRMS according to the 2017 McDonald revised criteria [14]. Eligible patients were those who had been receiving NTZ treatment for at least 6 months. The selection was based on clinical records indicating continuous NTZ therapy, either as SID or EID. The study was conducted following the principles of the Declaration of Helsinki. As a retrospective review of existing medical records, patient confidentiality was strictly maintained, with all data anonymized before analysis.

We performed a thorough review of the medical records of the included patients. The collected data included sociodemographic characteristics (age, sex, employment status, etc.), body mass index (BMI), details of NTZ treatment (duration, dosage, and frequency), previous MS therapies before initiating NTZ, and Expanded Disability Status Scale (EDSS) scores. EDSS scores were used to evaluate the disability status of patients at the time of their last visit. In addition, our review approach involved a thorough evaluation of potential adverse events linked to the use of NTZ, specifically emphasizing PML. As part of this assessment, we ensured that the serological status of the JCV was evaluated biannually for each patient.

Patients were grouped based on their NTZ dosing regimen. The SID group received NTZ every 4 weeks, whereas the EID group received it every 6 weeks. All patients were initially started on the standard interval dosing. Transitions to EID typically occurred after at least 2 years of treatment, primarily among patients who tested positive for anti‐JCV antibodies, in alignment with routine clinical practice aimed at reducing the risk of PML. Treatment regimen decisions were made at the discretion of the treating neurologist, considering clinical stability, JCV serostatus, and patient preference.

Treatment adherence was determined based on the timing of NTZ administration; any treatment administered 1 week or later than the scheduled time was classified as nonadherence. We examined differences in treatment duration, age, rate of treatment nonadherence, and clinical and radiological disease activity. In this study, clinical activation was defined as the occurrence of relapse. A relapse is characterized by new or worsening neurological symptoms lasting for more than 24 h and not attributable to other causes, such as infection or fever. Radiological activation is assessed through magnetic resonance imaging (MRI) and is defined as the presence of new or enlarging T2 hyperintense lesions or new gadolinium‐enhancing lesions on brain MRI. The MRI protocol included T2‐weighted, T1‐weighted, FLAIR, and gadolinium‐enhanced sequences, adhering to established guidelines for MS imaging. The presence of new or enlarging T2 lesions or gadolinium‐enhancing lesions indicates active inflammation and demyelination in the central nervous system, characteristic of MS disease activity. Every MR image is assessed by two neurologists, and in cases of ambiguity, a second opinion from a specialist in neuroinflammatory disorders is sought to confirm the findings.

The primary outcomes of the study were clinical and radiological disease activity and treatment nonadherence rates. Disease activity was assessed on the basis of clinical records, which included neurological assessments and MRI findings.

### 2.2 Statistical Analysis

Patient data collected in the study were analyzed using IBM SPSS Statistics for macOS, version 29.0 (IBM Corp., Armonk, NY). Categorical variables are presented as frequencies and percentages, while continuous variables are expressed as medians with minimum and maximum values. The Shapiro–Wilk test was applied to assess the normality of continuous data distributions. Since the data did not follow a normal distribution, non‐parametric statistical tests were used accordingly. The Mann–Whitney *U* test was employed for comparisons of continuous variables between groups. Categorical variables were analyzed using the chi‐square test or Fisher's exact test, as appropriate. In addition to the primary analyses, we performed further statistical tests to account for the sex imbalance between groups. A two‐way analysis of variance (ANOVA) was conducted to assess whether baseline EDSS scores were influenced by treatment group, sex, or their interaction. Logistic regression analysis was also performed to evaluate the impact of group assignment and sex on the likelihood of clinical worsening. A *p*‐value of < 0.05 was considered statistically significant.

## 3 Results

In this retrospective analysis, we evaluated 80 patients with RRMS who were treated with NTZ via either the SID or the EID. Patients were grouped as 52 receiving SID (300 mg every 4 weeks) and 28 receiving EID (300 mg every 6 weeks). Demographic characteristics showed significant differences in sex distribution (78.8% female in the SID group vs. 46.4% in the EID group; *p* = 0.007), although no significant differences were found in median age (32 years in the SID group vs. 36 years in the EID group; *p* = 0.209). Patients in the EID group had a longer median duration of NTZ (35 months vs. 12 months in the SID group; *p* < 0.001; Table 1).

**TABLE 1:** Demographic and clinical characteristics of patients by natalizumab dosing regimen (SID vs. EID).

Variables	SID (*n* = 52)	EID (*n* = 28)	*p* *
Sex			
Male	11 (21.2)	15 (53.6)	0.007
Female	41 (78.8)	13 (46.4)	
BMI	24.1 (17.4–36.3)	24.5 (18–31.2)	0.276
Age (years)	32 (19–62)	36 (21–60)	0.209
Education			
Primary‐secondary education	10 (19.2)	6 (21.4)	0.214
High school	23 (44.2)	7 (25)	
University‐Graduate education	19 (36.5)	15 (53.6)	
Occupation			
Unemployed	15 (28.8)	1 (3.6)	0.081
Homemaker	15 (28.8)	11 (39.3)	
Private Sector Employee	13 (25)	8 (28.6)	
Public Sector Employee	6 (11.5)	7 (25)	
Retired	1 (1.9)	1 (3.6)	
Student	2 (3.8)	0 (0)	
Comorbidity			
None	41 (78.8)	22 (78.6)	0.383
Hypertension	8 (15.4)	3 (10.7)	
Diabetes mellitus	1 (1.9)	0 (0)	
Coronary artery disease	1 (1.9)	0 (0)	
Thyroid disease	1 (1.9)	1 (3.6)	
Other autoimmune diseases	0 (0)	2 (7.1)	
Number of comorbidities			
None	41 (78.8)	22 (78.6)	0.537
1	9 (17.3)	6 (21.4)	
2	2 (3.8)	0 (0)	
Number of drugs used			
0	1 (1.9)	0 (0)	0.329
1	19 (36.5)	5 (17.9)	
2	22 (42.3)	16 (57.1)	
3	5 (9.6)	5 (17.9)	
> 3	5 (9.6)	2 (7.1)	

*Note:* Values are presented as *n* (%) or median (min–max), unless otherwise indicated.

Abbreviations: BMI, body mass index; EID, extended interval dosing; SID, standard interval dosing.

* *p* values reflect statistical significance, where *p* < 0.05 is considered significant.

MS symptom onset and treatment history were also evaluated. The proportion of patients with monosymptomatic versus polysymptomatic disease onset was similar between the groups (57.7% monosymptomatic in SID vs. 57.1% in EID; *p* = 1.00). Additionally, no significant difference was observed in the age of onset of the first MS symptom (22.5 years in the SID group vs. 26 years in the EID group; *p* = 0.084) or the total number of disease‐modifying therapies (DMTs) used before switching to NTZ (*p* = 0.489; Table 2).

**TABLE 2:** Multiple sclerosis symptom characteristics and treatment history by natalizumab dosing regimen (SID vs. EID).

Variables	SID (*n* = 52)	EID (*n* = 28)	*p* *
MS symptom onset			
Monosymptomatic	30 (57.7)	16 (57.1)	1.00
Polysymptomatic	22 (42.3)	12 (42.9)	
Age of onset of first symptom	22.5 (12–56)	26 (13–50)	0.084
Total disease duration (from first symptom to last visit) (years)	1.5 (1–14)	1 (1–12)	0.274
Total number of DMT used before NTZ	1 (0–5)	1 (0–4)	0.489
The last treatment before NTZ			
Interferons	17 (35.4)	4 (14.8)	0.042
Glatiramer acetate	5 (10.4)	5 (18.5)	
Teriflunomide	4 (8.3)	6 (22.2)	
Dimethyl fumarate	11 (22.9)	2 (7.4)	
Fingolimod	8 (16.7)	10 (37)	
Azathioprine	1 (2.1)	0 (0)	
Rituximab	2 (4.2)	0 (0)	
Natalizumab treatment duration (SID) (months)	12 (6–120)	35 (10–84)	< 0.001
Number of doses in the NTZ SID regimen	12 (6–104)	24.5 (6–63)	0.037
Natalizumab treatment duration (EID) (months)		10 (6–36)	NA
Number of doses in the NTZ EID regimen		6.5 (4–23)	NA

*Note:* Values are presented as *n* (%) or median (min–max), unless otherwise indicated.

Abbreviations: DMT, disease‐modifying therapy; EID, extended interval dosing, MS, multiple sclerosis; NTZ, natalizumab, SID, standard interval dosing.

* *p* values reflect statistical significance, where *p* < 0.05 is considered significant.

Clinical and radiological disease activity showed no significant differences between the groups. Combined clinical and radiological worsening occurred in 9.6% of the SID patients and 17.9% of the EID patients (*p* = 0.308). Radiological worsening alone was observed in 5.8% of the SID patients compared with 7.1% of the EID patients (*p* = 1.00). Clinical worsening was similar between the groups (9.6% in the SID group vs. 10.7% in the EID group; *p* = 1.00). Disability status, as measured by EDSS scores, showed no significant differences between the two groups. The median current EDSS score was 1.5 (range: 0–7.5) in the SID group and 1.0 (range: 0–5) in the EID group (*p* = 0.251). Baseline EDSS prior to NTZ initiation was similar in both groups (median: 2.0 in both; *p* = 0.910; Table 3).

**TABLE 3:** Treatment adherence and clinical outcomes by natalizumab dosing regimen (SID vs. EID).

Variables	SID (*n* = 52)	EID (*n* = 28)	*p* *
Treatment adherence (SID)			
Compliant	43 (82.7)	23 (82.1)	1.00
Noncompliant	9 (17.3)	5 (17.9)	
Number of noncompliant doses in the last 6 months	1 (1–2)	1.5 (1–9)	0.291
Treatment adherence (EID)			
Compliant		24 (85.7)	NA
Noncompliant		4 (14.3)	
Reason for treatment discontinuation			
Disease activity	4 (33.3)	1 (20)	0.438
JCV positivity	6 (50)	2 (40)	
Treatment noncompliance	1 (8.3)	0 (0)	
Pregnancy	1 (8.3)	2 (40)	
Initial JCV positivity	19 (36.5)	12 (42.9)	0.754
Initial JCV index	1.6 (0.5–3.3)	0.8 (0.3–4.1)	0.152
Last JCV positivity	27 (51.9)	14 (50)	1.00
Last JCV index	2.1 (0.4–4.2)	1.7 (0.3–4.3)	0.362
EDSS before NTZ	2 (0–5)	2 (0–5)	0.910
EDSS when starting to use EID		1 (0–5)	NA
Current EDSS	1.5 (0–7.5)	1 (0–5)	0.251
Clinical + Radiological worsening	5 (9.6)	5 (17.9)	0.308
Radiological worsening	3 (5.8)	2 (7.1)	1.00
Clinical worsening	5 (9.6)	3 (10.7)	1.00

Abbreviations: EDSS, Expanded Disability Status Scale; EID, extended interval dosing; JCV, John Cunningham virus; NA, not applicable, measured only in EID group; NTZ, natalizumab; SID, standard interval dosing.

* *p* values reflect statistical significance, where *p* < 0.05 is considered significant.

Adherence rates to treatment were comparable between the groups, with 82.7% adherence in the SID group and 85.7% in the EID group (*p* = 1.00). There were no significant differences in the reasons for treatment discontinuation, including disease activity, JCV positivity, or pregnancy (Table 3). Importantly, no cases of PML were reported during the study period.

Given the observed sex imbalance between treatment groups, we conducted additional analyses to evaluate the potential prognostic impact of sex on baseline characteristics and outcomes. No significant differences were found between male and female patients in terms of baseline EDSS scores prior to natalizumab initiation (median EDSS: male 2.0 vs. female 2.0; *p* = 0.566). Moreover, sex was not significantly associated with combined clinical and radiological worsening rates during the follow‐up period (males 11.5% vs. females 13.0%; *p* = 1.000). A two‐way ANOVA confirmed that there was no significant interaction between treatment group and sex in relation to EDSS scores (*p* = 0.957). Logistic regression analysis indicated that neither treatment group nor sex was a significant predictor of clinical worsening (*p* = 0.240 and *p* = 0.582, respectively).

## 4 Discussion

Our study offers valuable insights into the comparative efficacy and safety of EID versus SID of natalizumab in patients with RRMS. The analysis revealed similar rates of combined clinical and radiological worsening, as well as radiological and clinical worsening, between the two groups. Notably, the adherence rates were comparable across both groups, and no cases of PML were observed.

Natalizumab remains a particularly valuable option in the management of RRMS due to its high efficacy in preventing relapses and radiological disease activity, while lacking lymphocyte‐depleting properties, unlike other high‐efficacy disease‐modifying therapies such as anti‐CD20 monoclonal antibodies or alemtuzumab [15]. This characteristic allows for rapid immune reconstitution upon treatment discontinuation, making it a favorable option for patients who may require flexibility in therapy planning or are concerned about long‐term immunosuppression.

One of the most important findings is the absence of significant differences in clinical and radiological worsening between the two dosing regimens, reinforcing the notion that EID is a viable and effective alternative to SID for many patients. This finding is consistent with previous research, including the NOVA trial, which demonstrated that EID maintains similar efficacy to SID in terms of clinical relapses and MRI outcomes, with minimal differences in adverse events [16, 17, 18, 19, 20]. Our results further support these findings, showing that extending the dosing interval does not compromise clinical or radiological disease control. Additionally, our study highlights the “clinico‐radiological paradox,” wherein clinical symptoms and radiological findings may not always correlate [21, 22]. Both dosing regimens showed low and comparable rates of clinical and radiological worsening, emphasizing the importance of a holistic approach to disease management that considers both clinical outcomes and imaging markers [16]. This finding is consistent with the results of other studies, which have shown that clinical stability can be maintained even with extended dosing intervals [16, 17, 18, 19, 20].

Male sex has been consistently associated with a more severe disease course in MS, including faster disability accumulation, greater cognitive impairment, and increased risk of secondary progression [23, 24]. Given the sex imbalance observed in our study (greater male representation in the EID group), one might anticipate poorer outcomes for EID‐treated patients. However, our additional analyses demonstrated no statistically significant sex‐related differences in baseline disability (EDSS) or clinical and radiological disease activity during treatment. Two‐way ANOVA demonstrated that EDSS scores before natalizumab were not significantly influenced by sex or treatment group. Furthermore, logistic regression showed that neither treatment group nor sex was associated with clinical worsening. This suggests that the therapeutic effectiveness of natalizumab's extended dosing interval is maintained, even in patient populations with potentially worse prognostic demographics. This observation aligns with prior literature indicating that natalizumab efficacy is robust across various patient subgroups [9, 19].

Our study challenges the assumption that EID might lead to poorer treatment adherence [10]. We found no significant difference in adherence rates between the SID and EID groups. These findings suggest that adherence is influenced by factors beyond the dosing schedule (e.g., patient education, support systems, individual preferences), challenging the assumption that less frequent dosing inherently leads to lower compliance [8, 20]. Maintaining consistent treatment schedules remains essential for therapeutic efficacy in MS, and our findings underscore the need for a comprehensive approach to patient compliance, incorporating elements such as patient education, support systems, and personalized treatment planning [25, 26].

The established efficacy of monthly NTZ in managing MS is moderated by the risk of PML, particularly in patients with prolonged treatment and JCV seropositivity [1, 4, 5]. Our findings are consistent with those of previous studies, indicating that EID maintains efficacy while potentially reducing the risk of PML. Furthermore, the absence of PML cases and the similar safety profiles observed in both dosing regimens highlight EID as a promising option for patients with RRMS [17, 20]. In addition to EID, interval therapy involving planned annual treatment interruptions has also been explored as an alternative strategy. Berkovich et al. demonstrated that an annual 12‐week interruption of NTZ treatment was not associated with clinical or radiological worsening and was accompanied by a reversible decrease in elevated peripheral CD4+ T‐cell counts induced by natalizumab. These findings highlight the pharmacodynamic reversibility of NTZ and support further consideration of flexible treatment approaches in selected patients, particularly those at higher risk of PML [13].

Our study has several limitations. First, as a retrospective analysis, the study design inherently limits the ability to establish causal relationships, and the data relied on clinical records, which may introduce bias or missing information. Additionally, the relatively small sample size, especially in the EID group, may limit the generalizability of the findings. Another limitation of this study is the significant sex imbalance between the treatment groups. Although male sex has been associated with worse MS outcomes, our additional analyses (two‐way ANOVA and logistic regression) showed that neither sex nor treatment group significantly influenced baseline EDSS scores or clinical worsening. These findings suggest minimal impact of sex‐related bias in this cohort; however, future prospective studies with balanced group demographics and advanced statistical matching methods remain essential to validate these observations. Furthermore, the marked difference in NTZ treatment durations between the groups presents another limitation. Patients in the EID group had substantially longer exposure to the drug, which may have influenced adherence, efficacy, and safety outcomes. These differences should be considered when interpreting group comparisons. Moreover, the longer follow‐up time in the EID group could have increased the likelihood of detecting clinical or radiological worsening. Therefore, the observation that worsening rates remained similar between groups may support the sustained efficacy of EID over extended treatment periods, although this should be interpreted with caution due to the non‐randomized design. Larger multicenter prospective studies are needed to confirm these results. Another limitation is the relatively short follow‐up period, which may not capture long‐term outcomes or rare adverse events such as PML. Furthermore, this study did not assess patient‐reported outcomes, which are critical for understanding the broader impact of treatment on quality of life and symptom management. Despite these limitations, our study has several strengths. This is one of the few multicenter analyses comparing the EID and SID of NTZ in the RRMS. The inclusion of clinical and radiological measures provides a comprehensive assessment of disease activity. Moreover, the study highlights the comparable safety profiles and treatment adherence between the two regimens, contributing to the growing evidence that EID can be a viable alternative to SID in RRMS management. The absence of PML cases in both groups also underscores the safety of NTZ, particularly when an extended dosing schedule is used.

In conclusion, our findings highlight the potential of EID as a safe and effective alternative to SID in RRMS management. The comparable efficacy, adherence, and safety profiles between the two dosing regimens reinforce the importance of individualized treatment strategies. However, these findings should be interpreted with caution due to several study limitations, including the retrospective design, small sample size (particularly in the EID group), and baseline imbalances in sex distribution and treatment duration. Future research should continue to explore the long‐term outcomes of EID, with a focus on optimizing dosing regimens to balance efficacy, safety, and patient convenience.

## Ethics Statement

The procedures used in this study adhere to the tenets of the Declaration of Helsinki. Informed consent was obtained from the patients included in the study. We confirm that we have read the Journal's position on issues involved in ethical publication and affirm that this report is consistent with those guidelines.

## Conflicts of Interest

The authors declare no conflicts of interest.

## Data Availability

The data that support the findings of this study are available on request from the corresponding author. The data are not publicly available due to privacy or ethical restrictions.
